# The Iconography of Vermin

**DOI:** 10.3201/eid1902.AC1902

**Published:** 2013-02

**Authors:** Polyxeni Potter

**Affiliations:** Author affiliation: Centers for Disease Control and Prevention, Atlanta, Georgia, USA

**Keywords:** art science connection, emerging infectious diseases, art and medicine, Georges de La Tour, The Flea Catcher, the iconography of vermin, fleas, plague, vector-borne infections, French painting, about the cover

**Figure:**
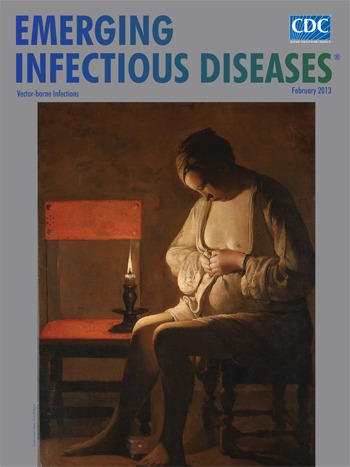
**Georges de La Tour (1593‒1652) *La Femme à la puce* (*The Flea Catcher*) (1638) Oil on canvas (90 cm × 120 cm)** Musée Lorrain, Nancy. Photo P. Mignot

“Haughty, sharp-tongued, self-assured, unbearably self-sufficient, stingy, and violent beyond measure,” is how Georges de La Tour was described by his contemporaries. Municipal records confirm that he refused to pay his share to feed the hungry during times of famine. He assaulted an officer, beat a peasant, and made himself obnoxious to everyone by “sending his dogs after hare into the standing crops which they trample down and ruin.”

A “painter” is how he described himself in the marriage contract in 1617. Shortly after the wedding, he moved to Lunéville, a prosperous town near Nancy, in Lorraine, now France, where he lived and worked. His early life and training remain otherwise ambiguous. He was influenced by the style of Caravaggio, either from travel to Italy or from contact with the Dutch followers of the Italian master. He found fame and fortune in his lifetime and was known as “Painter to the King.” He had 10 children, three of whom lived to adulthood. His son Étienne studied under him. La Tour died suddenly, possibly of the plague, within a few days of the deaths in his household of his wife and servant. He was soon forgotten to be discovered hundreds of years later and become an icon, anointed among the greats of his generation.

The 1630s was a turbulent period for Lorraine, a region contested by France and Germany for centuries. The 30 Years’ War and consequent epidemics, famine, and destruction, compounded by a fire in 1638 that burned Lunéville to the ground, contributed to the loss of much of La Tour’s legacy, as many as 400 works. A few remaining paintings were variously thought to be the work of Ribera, Zurbarán, Murillo, Velázquez, Rembrandt, and always, Caravaggio. Some paintings are still emerging from oblivion. *The Flea Catcher*, on this month’s cover, was not attributed to him until 1955.

In following Caravaggio, La Tour rejected Baroque classicism, the art movement of his age. He abandoned architectural backdrops and complicated scenes for solitary figures in dark tones. But his kinship with the master went beyond the dramatic chiaroscuro. The two shared, along with a troubling inability to cope with ordinary life, an incongruous gift for capturing its poetry on canvas. Well acquainted with darkness in the world, they brought into their paintings light. And though their choice of subjects wandered into the rogue—criminals, thieves, beggars—the light cast an aura of spirituality, driving the mood, the character, and the message.

In a departure from Caravaggio, La Tour introduced in his works the actual source of light, usually a candle, and became famous for his religious night scenes, often referred to as nocturnes. In these, he moved away from the traditional halos and wings, injecting an earthy holiness into his figures: Sebastian, patron saint of plague victims, pious women who nursed the wounded, and several versions of penitent Mary Magdalene. Alternating light and dark built mystery and stillness into these scenes, which, stripped of extraneous background, attained an almost geometric simplicity well ahead of the times.

*The Flea Catcher* has been unanimously accepted as the work of La Tour, although initially the subject matter caused confusion. Seventeenth-century art was filled with flea-searching figures painted by European masters. The Dutch often included daily bodily search for parasites in their repertoire, but flea and louse iconography was not part of French art. And although clearly in La Tour’s nighttime style, *The Flea Catcher* is different from his other works and from Dutch works on this subject. True to genre, the Dutch offerings were playful tongue-in -cheek, even erotic presentations, along the lines of, if not inspired by, poetry from Ovid to John Donne. “Madam, that flea, which crept between your breasts / I envied that there he should make his rest; / The little creature’s fortune was so good / That angels feed not on so precious food.”^1^

The phase of La Tour’s career during which *The Flea Catcher* was painted is not known, nor are the circumstances of naming the work. The dating is also approximate. Its execution, whether in the 1630s or 1640s, did coincide with instances of the plague in Lorraine and with troop movements through the region. But the cause of plague was unknown. Flea hunting was a mindless sport intended to provide relief from annoying bites and itching. And, of course, there was the issue of cleanliness.

The Dutch were notorious advocates of cleanliness. Gerard ter Borch and others often paid tribute to the dictum “spiritual purity starts with a clean body.” A woman was considered the “moral laundress” of the household and the guardian of proper child care: “Lazy mother, lousy heads.” Although French artists were not under as much pressure as the Dutch to couch moral messages in genre scenes, a moral or spiritual interpretation of La Tour’s woman’s search for fleas is intriguing. Certainly morality is present in the background of other well-known La Tour paintings (*The Fortune Teller, The Card Sharp with the Ace of Clubs*). In *The Flea Catcher,* the woman’s focus on the task rivals any Dutch example. And whatever it may have lost from abandoning the lighthearted approaches preceding it, this work made up in silence and intensity.

Unlike La Tour’s other work, mostly religious and genre scenes, *The Flea Catcher* has a complicated, even mysterious, aura. Not so much in its intimacy and introspection, which are found in most all his work, but in the discrepancy between these and the mundane task described. The enigmatic nature of the painting has attracted multiple interpretations. Some observers view the flea crushing as ancillary to the quiet contemplation and sadness implicit in the figure’s posture. Others take a religious approach. They see a fallen woman, possibly expecting a child, a penitent Magdalene pondering the excesses of her past life. Some viewers sense spiritual contemplation, a form of asceticism that comes from introspective concentration on a mundane task. And yet others suggest that the woman is not hunting fleas at all but praying the rosary or inspecting her garment, as the candlelight may be attracting fleas. Whatever the interpretation, the staging is pure La Tour: passive with an air of personal transcendence.

In this treatment of flea hunting, the viewer is given privileged access. The setting is sparse, fluid, linear, and intimate. The woman in plain wrap is in a stage of undress, her hair concealed, head downcast, bust and chest pathetically exposed, belly engorged. The hands, frozen in the all too familiar flea-crushing curl, are tightened to trap the undesirable guest between the thumbnails. In a characteristic maneuver, La Tour contrasts the impassive face with active, engaged hands. A smoking flame, the artist’s signature, exudes an otherworldly calm as it exposes red tones in the adjacent chair and settles lightly on the woman’s face. The emphasis here is not on charm but on drama, and the intense introspection implies deeper concerns.

Historical and religious references aside, La Tour’s *femme à la puce* begs for a contemporary interpretation, one informed by current knowledge of fleas, plague, and human frailty relieved by introspection. For as she absently tried to rid her body of fleas, La Tour’s woman became one more human protest against the ubiquitous pests. Whatever the distracting affliction she might have contemplated during her hunt, it would pale in the face of what she did not know about their behavior as vectors of disease.

Long a favorite of poets, the flea was an allusion to all sorts of mischief, a kind of literary pet. “If you turn me into anything, let it be in the likeness of a little pretty frisking flea that I might be here and there and everywhere.”^2^ Far from its reputation during La Tour’s lifetime as a harmless, even amusing insect, the flea has since emerged as enemy of public health, no less from its role in the spread of plague, a most dreaded scourge.

Fleas may be some of the most modern insects. Part of their success as vectors comes from their strong, but not absolute, preference for the blood of a good reservoir species (rodents, especially rats globally, prairie dogs and squirrels in the United States), along with a willingness to feed on humans when opportunity and need arise. Fleas that prefer prairie dog blood will feed on and infect a person who wanders through a prairie dog village after a plague-associated prairie dog die off. This less than desirable feed transforms an unrecognized sylvatic cycle into human illness and death. Fleas have demonstrated admirable staying power, causing pandemics, beginning in antiquity, and maintaining to this day a sylvatic cycle that bubbles along with few or no human cases but does not go away.

What does La Tour’s downcast woman have to do with modern fleas? She is collateral damage to a natural cycle of infection that engages rodent reservoir hosts and the flea vectors. Whatever her emotional situation, it probably had less to do with her worth as a person than with her status in the world, as misfortune generally does. She is in some way as much a host of her calamity as of the flea between her thumbs.

## Footnotes

1 “On a Flea on His Mistress’ Bosom,” ascribed to John Donne.

2 *Doctor Faustus*. Christopher Marlowe (1588‒1592).
